# Complete vaccination coverage of children born in 2017-2018, living in urban areas of state capitals and in 12 inland cities in Brazil: a population-based survey from a retrospective cohort study

**DOI:** 10.1590/S2237-96222024v33e20231101.especial2.en

**Published:** 2024-11-01

**Authors:** José Cássio de Moraes, Ana Paula França, Ione Aquemi Guibu, Rita Barradas Barata, Carla Magda Allan Santos Domingues, Maria da Gloria Teixeira, ICV 2020 Group, Adriana Ilha da Silva, Alberto Novaes Ramos, Ana Paula França, Andrea de Nazaré Marvão Oliveira, Antonio Fernando Boing, Carla Magda Allan Santos Domingues, Consuelo Silva de Oliveira, Ethel Leonor Noia Maciel, Ione Aquemi Guibu, Isabelle Ribeiro Barbosa Mirabal, Jaqueline Caracas Barbosa, Jaqueline Costa Lima, José Cássio de Moraes, Karin Regina Luhm, Karlla Antonieta Amorim Caetano, Luisa Helena de Oliveira Lima, Maria Bernadete de Cerqueira Antunes, Maria da Gloria Teixeira, Maria Denise de Castro Teixeira, Maria Fernanda de Sousa Oliveira Borges, Rejane Christine de Sousa Queiroz, Ricardo Queiroz Gurgel, Rita Barradas Barata, Roberta Nogueira Calandrini de Azevedo, Sandra Maria do Valle Leone de Oliveira, Sheila Araújo Teles, Silvana Granado Nogueira da Gama, Sotero Serrate Mengue, Taynãna César Simões, Valdir Nascimento, Wildo Navegantes de Araújo

**Affiliations:** 1 Santa Casa de São Paulo, Faculdade de Ciências Médicas, São Paulo, SP, Brazil; 2 Consultora independente, Brasília, DF, Brazil; 3 Universidade Federal da Bahia, Instituto de Saúde Coletiva, Salvador, BA, Brazil; Universidade Federal do Espírito Santo, Vitória, ES, Brazil; Universidade Federal do Ceará, Departamento de Saúde Comunitária, Fortaleza, CE, Brazil; Faculdade Ciências Médicas Santa Casa de São Paulo, São Paulo, SP, Brazil; Secretaria de Estado da Saúde do Amapá, Macapá, AP, Brazil; Universidade Federal de Santa Catarina, SC, Brazil; Organização Pan-Americana da Saúde, Brasília, DF, Brazil; Instituto Evandro Chagas, Belém, PA, Brazil; Faculdade de Ciências Médicas Santa Casa de São Paulo, Departamento de Saúde Coletiva, São Paulo, SP, Brazil; Universidade Federal do Rio Grande do Norte, Natal, RN, Brazil; Universidade Federal do Ceará, Departamento de Saúde Comunitária, Fortaleza, CE, Brazil; Universidade Federal de Mato Grosso, Cuiabá, MT, Brazil; Universidade Federal do Paraná, Curitiba, PR, Brazil; Universidade Federal de Goiás, Goiânia, GO, Brazil; Universidade Federal do Piauí, Teresina, PI, Brazil; Universidade de Pernambuco, Faculdade de Ciências Médicas, Pernambuco, PE, Brazil; Instituto de Saúde Coletiva, Universidade Federal da Bahia, Salvador, BA, Brazil; Secretaria de Estado da Saúde de Alagoas, Maceió, AL, Brazil; Universidade Federal do Acre, Rio Branco, AC, Brazil; Universidade Federal do Maranhão, Departamento de Saúde Pública, São Luís, MA, Brazil; Universidade Federal de Sergipe, Aracaju, SE, Brazil; Secretaria Municipal de Saúde, Boa Vista, RR, Brazil; Fundação Oswaldo Cruz, Mato Grosso do Sul, Campo Grande, MS, Brazil; Fundação Oswaldo Cruz, Escola Nacional de Saúde Pública Sergio Arouca, Rio de Janeiro, RJ, Brazil; Universidade Federal do Rio Grande do Sul, Porto Alegre, RS, Brazil; Fundação Oswaldo Cruz, Instituto de Pesquisa René Rachou, Belo Horizonte, MG, Brazil; Secretaria de Desenvolvimento Ambiental de Rondônia, Porto Velho, RO, Brazil; Universidade de Brasília, Brasília, DF, Brazil

**Keywords:** Cobertura de vacunación, Encuestas Epidemiológicas, Programas de Inmunización, Retraso Vacunal, Vacunación, Vaccination Coverage, Epidemiological Surveys, Immunization Programs, Delayed Vaccination, Vaccination

## Abstract

**Objective:**

To estimate vaccination coverage in children born between 2017-2018, living in urban areas of state capitals, the Federal District and 12 inland municipalities in Brazil, and to identify associated factors.

**Methods:**

This was a household survey conducted between 2020-2022, among children up to 24 months old. Vaccination coverage was estimated according to family, maternal and child characteristics.

**Results:**

Among the 37,801 children in the sample, complete coverage (doses administered) was 60.1% (95%CI 58.6;61.6) and 6.1% (95%CI 5.4;7.0) had not received any vaccines. Coverage was lower among children of mothers with lower level of education (OR = 0.70; 95%CI 0.54;0.90) and in those who experienced delays in receiving any vaccine by 6 months old (OR = 0.28; 95%CI 0.24;0.32).

**Conclusion:**

Vaccination coverage is below the expected levels. Effective communication strategies are needed to reinforce the importance of routine vaccination, prevent delays and abandonment of the vaccination schedule, in order to recover the high coverage levels achieved in past decades.

## Study contributions

**Main results:**

Vaccination coverage with the complete schedule, excluding yellow fever, was 60% and lower among mothers with low education level and in children with delayed vaccination. With the exception of the intradermal BCG vaccine, the other vaccines did not reach the recommended levels.

**Implications for services:**

The vaccination process in primary healthcare centers can be improved by enabling the simultaneous administration of doses scheduled in a single session and implementing active search for absentees in order to reduce delayed vaccination and enhance access.

**Perspectives:**

The discussion of the results across the three levels of management can support proposals to improve the functioning of health services, especially the operational process in vaccination rooms, aimed at improving vaccination coverage.

## RESUMEN

**Objetivo:**

Estimar la cobertura vacunal en niños nacidos entre 2017-2018, residentes en áreas urbanas de las capitales, Distrito Federal y 12 municipios del interior de Brasil, e identificar factores asociados.

**Métodos:**

Encuesta de hogares realizada entre 2020-2022, en niños de hasta 24 meses. La estimativa de cobertura vacunal consideró las características familiares, maternas y del niño.

**Resultados:**

En los 37.801 niños de la muestra la cobertura completa (dosis aplicadas) fue del 60,1%, (IC_95_% 58,6;61,6) y el 6,1% (IC_95_% 5,4;7,0) no recibió ninguna vacuna. La cobertura fue menor en hijos de madres con menor escolaridad (OR = 0,70; IC_95_% 0,54;0,90) y en aquellos que tardaron en recibir alguna vacuna hasta los 6 meses de edad (OR = 0,28; IC_95_% 0,24;0,32).

**Conclusión:**

La cobertura vacunal está por debajo de las expectativas. Es necesario crear estrategias de comunicación efectivas para reforzar la importancia de la vacunación sistemática y volver a los altos niveles alcanzados en décadas pasadas.

## INTRODUCTION

Understanding the actual vaccination coverage of a community is crucial for proposing actions to improve or maintain the control of vaccine-preventable diseases.^1^ In the first two decades following the implementation of the National Immunization Program (*Programa Nacional de Imunizações – PNI*), in 1973, vaccination coverage remained low, but progressively reached optimal levels for the control, elimination or eradication of some vaccine-preventable diseases.^2^ However, since 2016 there has been a decline in coverage for all vaccines available in the PNI, which worsened during the COVID-19 pandemic years.^3^.^4^

The occurrence of epidemics, even with high coverage estimates, demonstrates the inaccuracy of these estimates, as revealed by household surveys. Coverage heterogeneity, not always accurately identified, poses a risk for accumulation of susceptible individuals, with the potential for the introduction and sustained circulation of infectious agents. The measles epidemic in 1997, which caught the epidemiological surveillance program by surprise, underscored the importance of accurate knowledge of vaccination coverage.^5^

In Brazil, vaccination coverage is monitored using estimates calculated from data generated by the PNI Information System (*Sistema de Informação do PNI – SI-PNI). The accuracy of these estimates is impacted by operational challenges that affect data quality and completeness, including underreporting from private vaccination services, local systems that are not integrated with the SI-PNI and deficiencies in technological structure and human resources for system management in health units.^2^*

Another factor contributing to inaccurate data recording in the SI-PNI is the number of vaccines in the current immunization schedule: 13 vaccines and 23 doses up to 24 months old. These vaccines are administered in over 36 thousand units of the Brazilian National Health System (*Sistema Único de Saúde – SUS*) across all Brazilian municipalities through various strategies, such as routine; coverage/focus block; indiscriminate or selective campaigns; house-to-house; schools, among others.^3^

In the 2007 vaccination coverage survey conducted in all Brazilian capitals in children aged 18 to 30 months, complete coverage with doses administered exceeded 80%.^5^ Periodically conducting surveys not only allows for more accurate estimates of these indicators, but also helps evaluate trends and identify aspects of living conditions that impact the vaccination process.^6^

The main objective of this survey was to estimate vaccination coverage among children born in 2017-2018, living in the urban areas of state capitals, the Federal District and in 12 inland municipalities in Brazil, and to identify factors associated with vaccination.

## METHODS

This was a population-based survey based on a retrospective cohort, conducted from 2020 to 2022, which verified compliance with the PNI vaccination schedule and the factors associated with vaccination.^7^

The study population was comprised of children born in 2017 and 2018, living in the urban areas of 26 capitals of the Federative Units (FU), the Federal District and in the following municipalities: Imperatriz/Maranhão state, Sobral/Ceará state, Caruaru/Pernambuco state and Vitória da Conquista /Bahia state (Northeast); Sete Lagoas/Minas Gerais state, Petrópolis/Rio de Janeiro state and Campinas/São Paulo state (Southeast); Londrina/Paraná state, Joinville/Santa Catarina state and Rio Grande/Rio Grande do Sul state (South); Rondonópolis/Mato Grosso state and Rio Verde/Goiás state (Midwest). The eligible municipalities had a population of over 100,000 inhabitants in 2020, were located outside the metropolitan region of their respective capitals and were selected by convenience.^7^

In the first stage, census tracts in each of the 39 municipalities were classified into four socioeconomic strata (A, B, C and D – with A being the best and D being the worst socioeconomic status). In each municipality, stratification cutoff points were different, taking into consideration the nominal income of the head of household, the percentage of heads of household with income above 20 times the minimum wage and the percentage of literate heads of household, using data from the 2010 demographic census.^7^

The set of municipalities studied was divided into 88 domains of interest (surveys), ranging from 1 to 4 per municipality, depending on the number of live births recorded on the Live Birth Information System in 2017 and 2018. The sample size calculation was based on a vaccination coverage of 70%, design effect of 1.4 and a 95% confidence level, resulting in 452 in each area of interest, totaling an expected sample size of 39,776 children.^7^ Sampling was conducted using systematic cluster sampling.

The complete research questionnaire consisted of nine blocks, however, for this article, variables related to the characteristics of the family (socioeconomic stratum, household consumption level, monthly household income and income transfer program), the child’s mother (education level and age group), the child (the healthcare service where they received the vaccine) and the vaccination process (coincidence of dates for vaccines recommended at 4 months old and delayed vaccination for any vaccine scheduled up to 6 months old) were analyzed. The household consumption level was defined according to criteria from the Brazilian Association of Research Companies.^8^

In order to calculate vaccination coverage, a photographic record of the vaccination booklet was obtained and for the child who did not have a booklet, the registration form from the SI-PNI was sought, and when it was not found in the system, the child was considered unvaccinated.^9^

A combination of different vaccines aimed at preventing the same diseases (e.g., triple bacterial vaccine [diphtheria, tetanus and pertussis, DTP], tetravalent, pentavalent or hexavalent for the respective diseases) was performed to accurately calculate coverage, taking into account vaccines administered by both public and private sectors.^9^

The difference between the dates recorded in the vaccination booklet and the child’s date of birth enabled the classification of the administered doses as valid and/or timely. Valid doses were those administered from 15 days before the date set by the PNI, respecting the minimum interval recommended for each dose. Timely doses were those administered between 15 days before and up to 30 days after the scheduled date.^3^.^7^

The indicator of complete vaccination coverage (up to 24 months old) was defined by considering the administration of all set of doses and boosters provided for in the official PNI schedule: BCG; hepatitis B (HepB); pentavalent (penta: DTP + haemophilus influenzae B + hepatitis B) – first + second + third doses; inactivated polio vaccine 1, 2 and 3 (IPV: first + second + third doses); human rotavirus vaccine (HRV: first + second doses); meningococcal C (MenC: first + second doses + booster); 10-valent pneumococcal vaccine (PCV-10: first + second doses); measles, mumps and rubella (MMR: first + second doses + booster); hepatitis A (HepA: first dose); chickenpox (VZV: single dose); attenuated oral poliovirus vaccine 1 and 3 (OPV: booster); diphtheria, tetanus and pertussis (DTP: booster). The yellow fever (YF-VAX) vaccine was excluded from the calculation, as it was not part of the routine schedule in some municipalities.^3,7^ For vaccines with multiple doses, the dropout rate was calculated using the following formula:


$$\left(\frac{\text{number of children who did not complete the schedule}}{\text{number of children who received the first dose}}\right)\times 100.$$

Delayed vaccination was defined as any dose administered 30 days after the date recommended in the PNI schedule.^7^

The coverage cascade for each vaccine was calculated using the number of vaccinated children in the numerator and, in the denominator, the number of children who received the immediately preceding dose of the vaccine. Thus, to calculate HepB vaccine coverage at birth, the denominator was the number of children who had received the BCG vaccine.

In order to identify differences in vaccination coverage among municipalities, the precision of the point estimate was assessed using 95% confidence intervals (95%CI). For the analysis of factors associated with vaccination coverage, odds ratios were estimated (OR) using logistic regression. Significant associations between variables and complete vaccination coverage in the crude analysis were adjusted for household income, maternal level of education and maternal age. All analyzes were performed using Stata, version 17, by means of *surve y module, taking into account the sample weights and the study design.*

The study was approved by the Human Research Ethics Committees of the Instituto de Saúde Coletiva da Universidade Federal da Bahia, opinion No. 3,366,818, on June 4, 2019, with Certificate of Submission for Ethical Appraisal (CAAE) 4306919.5. 0000.5030, and the Irmandade da Santa Casa de São Paulo, opinion No. 4.380.019, issued on November 4, 2020, with CAAE 39412020.0.0000.5479. 

## RESULTS

A total of 37,801 interviews (95.0%) were conducted across the 39 municipalities. The highest loss rate occurred in socioeconomic stratum A (16.2%) and there was no loss in strata C and D (table not shown).

The vaccine with the highest coverage (doses administered) was BCG, 89.8% (95% CI 88.6;90.8), while the lowest coverage rates were observed for the second dose of HRV, first booster dose of MenC and second dose of MMR vaccine, 82.2% (95%CI 80.9;83.5). Considering valid doses, the first booster of the PCV-10 had the lowest coverage: 73.8% (95%CI 72.0;75.4) (Table 1).

**Table 1:** Vaccination coverage of vaccines provided for in the National Immunization Program (PNI) calendar according to the classification of doses (applied, valid or timely) and ratio between coverage. Capital cities, Federal District and 12 municipalities in the interior of Brazil. National vaccination coverage survey, 2020 (n = 37,801)

**Vaccine**	**Complete coverage at 24 months**						**Valid/administered**	**Timely/administered**
	**Administered doses**		**Valid doses**		**Timely doses**			
	**%**	**(95% CI)**	**%**	**(95%CI)**	**%**	**(95%CI)**		
BCG	89.8	(88.6;90.8)	89.8	(88.6;90.8)	83.6	(82.3;84.8)	1.00	0.93
Hepatitis B (HepB)	88.8	(87.6;89.9)	88.8	(87.5;89.9)	85.7	(84.5;86.8)	1.00	0.97
Diphtheria, tetanus, pertussis , hepatitis B and *Haemophilus influenzae B* (penta – 3^rd^ dose)	88.1	(86.9;89.2)	87.5	(86.3;88.6)	56.3	(54.6;58.1)	0.99	0.64
Inactivated polio 1, 2 and 3 (IPV – 3^rd^ dose)	88.0	(86.8;89.1)	87.5	(86.3;88.6)	58.4	(56.6;60.0)	0.99	0.66
10-valent Pneumococcal (PCV-10 – 2^nd^ dose)	90.4	(89.4;91.4)	89.9	(88.8;90.9)	79.9	(78.5;81.1)	0.82	0.88
Human rotavirus (HRV – 2^nd^ dose)	82.2	([80.9;83.4)	75.8	(74.3;77.2)	66.2	(64.6;67.8)	0.92	0.81
Meningococcal C (MenC – 2^nd^ dose)	89.5	(88.4;90.4)	88.7	(87.6;89.7)	61.8	(60.1;63.4)	0.99	0.69
Hepatitis A (HepA)	88.2	(87.1;89.3)	87.0	(85.8;88.1)	49.9	(48.4;51.3)	0.99	0.57
Measles, mumps and rubella (MMR – 1^st^ dose)	90.9	(89.9;91.9)	90.0	(88.9;91.0)	52.3	(50.6;53.9)	0.99	0.58
Measles, mumps and rubella (MMR – 2^nd^ dose)	82.2	(80.9;83.5)	81.1	(79.8;82.3)	40.2	(38.8;41.7)	0.99	0.49
Chickenpox (VZV)	87.1	(85.9;88.1)	79.2	(77.6;80.7)	46.5	(45.0;48.0)	0.91	0.53
10-valent Pneumococcal (PCV-10 1^st^ booster)	85.0	(83.8;86.2)	73.8	(72.0;75.4)	43.9	(42.2;45.5)	0.87	0.52
Meningococcal C (MenC – 1^st^ booster)	82.2	(81.0;83.4)	76.6	(75.2;78.0)	42.0	(40.4;43.6)	0.93	0.51
Diphtheria, tetanus, pertussis (DTP – 1^st^ booster)	84.2	(83.1;85.3)	83.5	(82.3;84.6)	36.4	(34.9;38.0)	0.99	0.43
Attenuated oral polio 1 and 3 (OPV - 1^st^ booster)	86.2	(85.1;87.3)	81.8	(80.4;83.0)	43.1	(41.6;44.6)	0.95	0.50

Coverage with complete vaccination schedule (administered doses) for vaccines targeting diseases subject to eradication policy (polio) and regional elimination (measles and rubella) were, 88.0% (95%CI 86.8;89.1) and 82.2% (95%CI 80.9;83.5), respectively, both lower than the 95% proposed by the eradication/elimination plan (Table 1).

The greatest relative difference between doses administered and valid doses was observed for the second dose of PCV-10. All vaccines administered in the second year of life showed vaccination coverage below 50% for timely doses (Table 1).

Figure 1 shows the evolution of coverage according to the sequence outlined in the vaccination schedule. Among those who received the BCG vaccine (89.6%), only 60.1% completed the vaccination schedule, representing a significant drop (32.9%). In socioeconomic stratum A, BCG coverage was 83.1% (95%CI 78.6;87.2) and, by the end of follow-up, only 53.4% (95%CI 48.2;58.6) of children had received all vaccines. In stratum D, 90.7% (95%CI 89.2;92.3) of children received the BCG vaccine and the cascade ended with 60.8% (95%CI 58.7;62.8). The most significant drops were observed when the second dose of the HRV vaccine and the second dose of the MMR vaccine were administered.

**Graph 1:**
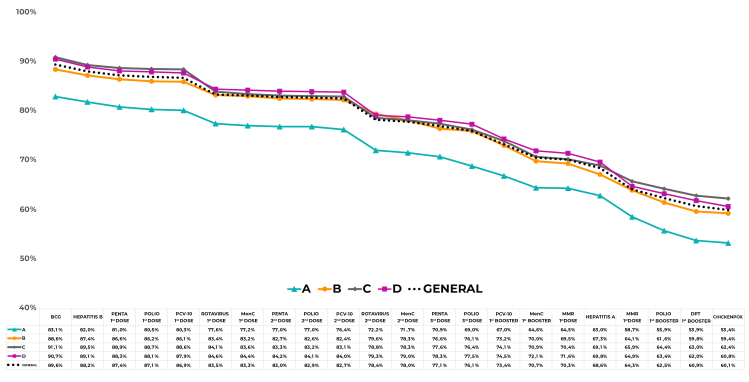
Cascade of doses applied of vaccines provided for in the PNI calendar, according to socioeconomic stratum. Capital cities, Federal District and 12 municipalities in the interior of Brazil. National vaccination coverage survey, 2020 (n = 37,801)

Across all municipalities, the dropout rates (doses administered) for the pentavalent vaccine (penta) were 4.0% and 1.4% for IPV. For the HRV, PCV-10 and MenC vaccines, the rates were 6.5%, 2.2% and 2.8%, respectively. For the MMR vaccine, the dropout rate was 9.6% (Figure 1).

Complete coverage for all municipalities studied (capitals + inland cities) was 60.1% (95% CI 58.6;61.6) for doses administered and 43.5% (95%CI 41.9;45, 2) for valid doses, representing a 27.6% difference (Table 2). Complete coverage for timely doses was extremely low, at 9.8% (95%CI 8.8;10.9), with an 83.7% difference from doses administered. The percentage of children who did not receive any doses of vaccine was 6.1% (95%CI 5.3;6.9).

**Table 2:** Complete vaccination coverage up to 24 months according to municipality and classification of doses (applied, valid and timely). Capital cities, Federal District and 12 municipalities in the interior of Brazil. National vaccination coverage survey, 2020 (n = 37,801)

**Municipality**	**Complete coverage at 24 months old**						**Valid / administered**	**Timely / administered**
	**Administered doses**		**Valid doses**		**Timely doses**			
	**%**	**(95%CI)**	**%**	**(95%CI)**	**%**	**(95%CI)**		
Porto Velho	64.8	(57.9;71.2)	54.0	(46.7;61.1)	8.9	(5.4;14.4)	0.83	0.14
Rio Branco	60.8	(53.2;67.8)	43.3	(38.0;48.6)	6.1	(2.6;13.6)	0.71	0.10
Manaus	54.1	(49.6;58.1)	42.9	(39.0;46.9)	3.2	(2.0;4.8)	0.79	0.06
Boa Vista	60.0	(48.7;70.3)	51.8	(39.6;63.8)	9.7	(6.0;15.4)	0.86	0.16
Belém	57.5	(47.4;67.0)	34.7	(24.9;46.0)	4.8	(2.0;10.8)	0.60	0.08
Macapá	35.8	(28.1;44.4)	25.8	(17.8;35.8)	4.2	(2.1;8.1)	0.72	0.12
Palmas	67.5	(60.5;73.8)	59.3	(51.5;66.2)	5.6	(2.9;10.5)	0.88	0.08
Imperatriz	46.3	(40.9;51.7)	34.2	(28.7;40.1)	4.1	(2.1;6.1)	0.74	0.09
São Luís	51.6	(43.0;60.1)	36.2	(27.4;46.1)	2.8	(1.4;5.1)	0.70	0.05
Teresina	73.7	(63.0;82.1)	64.6	(56.3;72.2)	10.2	(6.0;16.6)	0.88	0.14
Fortaleza	54.0	(47.3;60.6)	45.3	(39.1;51.7)	11.1	(8.0;15.2)	0.84	0.21
Sobral	57.7	(47.2;67.5)	47.1	(37.5;57.0)	17.4	(11.0;26.5)	0.82	0.30
Natal	36.6	(26.7;47.3)	31.5	(22.8;41.6)	5.3	(2.1;12.5)	0.86	0.14
João Pessoa	42.6	(36.3;49.2)	33.4	(27.2;40.2)	4.6	(3.3;6.4)	0.78	0.11
Caruaru	68.8	(60.1;76.3)	57.7	(49.4;65.7)	11.9	(7.8;17.9)	0.84	0.17
Recife	56.9	(49.6;63.9)	42.4	(34.9;50.2)	8.0	(5.4;11.9)	0.75	0.14
Maceió	58.3	(50.2;66.0)	42.2	(35.2;49.5)	7.5	(4.4;12.5)	0.72	0.13
Aracaju	65.3	(58.6;71.4)	52.4	(47.4;57.3)	15.1	(11.6;19.5)	0.80	0.23
Salvador	64.9	(60.5;69.1)	52.6	(47.1;58.1)	14.8	(9.5;22.3)	0.81	0.23
Vitória da Conquista	61.7	(51.2;71.1)	54.9	(43.5;65.9)	11.8	(5.7;22.9)	0.89	0.19
Belo Horizonte	63.8	(59.5;67.9)	46.2	(40.2;52.3)	13.2	(9.9;17.4)	0.72	0.21
Sete Lagoas	79.1	(75.1;82.7)	61.6	(53.6;69.1)	19.5	(15.2;24.6)	0.78	0.25
Vitória	57.1	(50.6;63.4)	34.3	(22.9;47.7)	6.7	(4.2;10.9)	0.60	0.12
Petrópolis	69.4	(61.0;76.8)	50.3	(34.4;66.1)	7.2	(4.3;11.7)	0.72	0.10
Rio de Janeiro	51.7	(45.8;57.4)	34.6	(29.9;39.7)	11.9	(8.9;15.7)	0.67	0.23
Campinas	63.0	(54.2;71.1)	43.3	(35.8;51.1)	11.1	(8.1;15.0)	0.69	0.18
São Paulo	64.0	(60.1;67.7)	44.2	(39.2;49.4)	13.1	(10.2;16.7)	0.69	0.20
Curitiba	74.4	(66.3;81.1)	49.2	(39.8;58.7)	6.6	(3.2;12.6)	0.66	0.09
Londrina	66.6	(50.5;79.6)	57.7	(41.9;72.1)	17.6	(8.7;32.3)	0.87	0.26
Joinville	71.4	(65.3;76.9)	58.4	(52.5;64.0)	11.4	(7.6;16.5)	0.82	0.16
Florianópolis	49.6	(40.8;58.5)	25.5	(19.0;33.4)	4.0	(2.1;7.5)	0.51	0.08
Porto Alegre	65.2	(59.7;70.3)	48.4	(42.8;54.0)	7.9	(5.4;11.5)	0.74	0.12
Rio Grande	57.2	(47.7;66.2)	37.8	(20.7;58.5)	11.3	(5.6;21.2)	0.66	0.20
Campo Grande	54.2	(48.2;60.1)	40.9	(35.9;46.2)	5.3	(3.7;7.6)	0.75	0.10
Cuiabá	60.9	(53.2;68.1)	46.8	(40.1;53.6)	5.3	(2.5;11.0)	0.77	0.09
Rondonópolis	44.9	(36.1;54.1)	36.9	(28.4;46.3)	8.5	(5.1;14.0)	0.82	0.19
Goiânia	56.7	(50.2;62.9)	47.9	(41.5;54.4)	10.6	(6.1;17.9)	0.84	0.19
Rio Verde	38.5	(30.4;47.3)	28.7	(23.5;34.5)	5.2	(2.3;11.5)	0.75	0.14
Brasilia	73.1	(69.3;76.6)	55.2	(50.5;59.8)	15.8	(12.8;19.5)	0.76	0.22
Capitals (n = 31,001)	59.9	(58.3;61.5)	43.1	(41.3;44.9)	9.7	(8.6;10.9)	0.72	0.16
Inland municipalities (n = 6,800)	62.3	(58.9;65.5)	48.0	(44.3;51.7)	11.0	(9.1;13;3)	0.77	0.18
Total (n = 37,801)	60.1	(58.6;61.6)	43.5	(41.9;45.2)	9.8	(8.8;10.9)	0.72	0.16

Complete coverage with administered, valid or timely doses was heterogeneous across the 39 municipalities studied (Table 2). Sete Lagoas/Minas Gerais state showed the highest complete coverage for administered, valid and timely doses. The lowest coverage for doses administered were found in Natal/Rio Grande do Norte state, for valid doses in Rio Verde/Goiás state and, for timely doses, in Manaus/Amazonas state. The greatest differences between valid and administered dose coverage (40.0%) were observed in Belém/Pará state and Vitória/Espírito Santo state. Regarding timely doses, the greatest difference in vaccination coverage was found in São Luís/Maranhão state (95.0%).

Five municipalities had complete coverage above 70% for doses administered (Teresina/Piauí state, Sete Lagoas/Minas Gerais state, Curitiba/Paraná state, Joinville/Santa Catarina state and Brasília/Distrito Federal), while seven showed coverage below 50% (Macapá/Amapá state, Imperatriz/ Maranhão state, Natal/Rio Grande do Norte state, João Pessoa/Paraíba state, Florianópolis/Sanata Catarina state, Rondonópolis/Mato Grosso state and Rio Verde/Goiás state). Taking into consideration only valid doses, only two municipalities (Teresina/Piauí state and Sete Lagoas/Minas Gerais state) showed coverage greater than 60%. With regard to timely doses, no municipality had coverage greater than 20%. In the North region, the municipality with the highest coverage (administered and valid doses) was Palmas/Tocantins state; in the Northeast region, Teresina /Piauí; in the Southeast region, Sete Lagoas/Minas Gerais state; in the South region, Joinville/Santa Catarina state and, in the Midwest region, Brasília/Distrito Federal (Table 2).

The average number of visits to vaccination rooms to complete the schedule was 10.8, three more than recommended by the PNI, and 95% of children who completed the schedule required up to 14 visits (table not shown).

The delay in administering vaccines at birth was 6.4% (95%CI 5.6;7.3). At 4 months old, when four vaccines are recommended, 21.5% (95%CI 20.2;22.8) of children were delayed in receiving at least one vaccine. In the first 6 months of life, 51.4% (95%CI 49.6;53.3) experienced delays in receiving at least one vaccine. The delay in the first dose of the MMR vaccine, which should be administered at 12 months, was 45.9% (95%CI 44.3;47.5) (table not shown).

Table 3 shows the complete coverage according to the presence of delayed vaccination. Coverage was lower for children who experienced delays in receiving any vaccines up to 6 months old: 56.7% (95%CI 54.8;58.6), compared to those without delays: 82.7 % (95%CI 80.5;84.8). The delay in administering vaccines at birth resulted in a relative difference of 17.4% in complete coverage. A similar difference was observed when the delay occurred in any of the vaccines administered at 4 months old. Any delay by 6 months old resulted in a 31.4% difference in complete coverage.

**Table 3:** Complete vaccination coverage up to 24 months of doses administered according to the occurrence of delay in at least one vaccine according to the period scheduled for vaccine administration. Capital cities, Federal District and 12 municipalities in the interior of Brazil. National vaccination coverage survey, 2020 (n = 37,801)

Recommended period for administration according to the vaccination schedule	Complete vaccination coverage				
	**Delayed vaccination**				**Difference attributed to delay** ^a^ **%**
	**No**		**Yes**		
	**%**	**(95%CI)**	**%**	**( 95%CI)**	
At birth	69.0	(67.5;70.4)	57.0	(50.9;62.8)	17.4
At 2 months	72.3	(70.8;73.7)	48.6	(44.3;53.0)	32.8
At 3 months	70.4	(68.9;71.9)	41.8	(38.6;45.1)	40.6
At 4 months	77.4	(75.8;78.9)	64.5	(61.7;67.2)	16.7
At 5 months	73.3	(71.6;75.0)	53.4	(51.1;55.7)	27.1
At 6 months	75.2	(73.3,76.9)	59.9	(57.6;62.2)	20.3
Up to 6 months	82.7	(80.5;84.8)	56.7	(54.8;58.6)	31.4

a) [(coverage without delay – coverage with delay) /coverage without delay x 100].

Vaccination coverage (doses administered) did not differ between children who received all vaccines exclusively in the public sector and those who received at least one vaccine in the private sector: 60.2% (95%CI 58.5;61.9) and 60.0% (95%CI 56.3;63.7), respectively.

**Table 4:** Complete vaccination coverage of doses administered up to 24 months and crude and adjusted odds ratios (OR) according to characteristics of families, mothers and children. Capital cities, Federal District and 12 municipalities in the interior of Brazil. National vaccination coverage survey, 2020 (n = 37,801)

Variables				**Crude analysis**		**Adjusted analysis**	
	**%**	**(95%CI)**		**OR**	**(95%CI)**	**OR**	**(95%CI)**
**Socioeconomic stratum**							
A	53.4	(48.2;58.6)		0.74	(0.58;0.93)	0.68	(0.53;0.87)
B	59.4	(54.9;63.8)		0.95	(0.77;1.16)	0.86	(0.69;1.07)
C	62.4	(60.0;64.8)		1.07	(0.94;1.23)	1.02	(0.88;1.19)
D	60.8	(58.7;62.8)		1.00		1.00	
**Household consumption level**							
A	60.3	(51.9;68.0)		1.10	(0.78;1.56)	0.88	(0.55;1.42)
B	60.8	(57.1;64.3)		1.13	(0.95;1.34)	1.05	(0.81;1.35)
C	63.4	(60.9;65.8)		1.26	(1.10;1.44)	1.16	(1.00;1.36)
D	57.8	(55.7;59.9)		1.00		1.00	
**Monthly household income (BRL)**							
≤ 1,000	58.1	(55.3;60.8)		1.00		1.00	
1,001 to 3,000	61.6	(59.2;64.0)		1.16	(1.00;1.35)	1.09	(0.93;1.29)
3,001 to 8,000	65.5	(62.1;68.7)		1.37	(1.14;1.64)	1.17	(0.95;1.44)
≥ 8,001	59.9	(54.2;65.4)		1.08	(0.83;1.40)	0.88	(0.66;1.18)
**Maternal education**							
No education/incomplete fundamentals	53.5	(49.5;57.3)		0.75	(0.61;0.92)	0.70	(0.54;0.90)
Complete fundamental	58.9	(55.0;62.6)		0.93	(0.76;1.14)	0.90	(0.70;1.17)
Full medium	62.4	(60.3;64.5)		1.08	(0.93;1.26)	0.98	(0.82;1.18)
Complete higher education	60.6	(57.5;63.6)		1.00		1.00	
**Maternal age group**							
< 20 years	51.6	(43.3;59.9)		1.00		1.00	
20 to 34 years old	59.5	(57.5;61.4)		1.37	(0.97;1.95)	1.26	(0.82;1.92)
35 years or older	61.4	(58.9;63.8)		1.49	(1.05;2.11)	1.29	(0.78;2.14)
**Income transfer program**							
Yes	59.5	(56.9;62.0)		1.00		1.00	
No	60.3	(58.4;62.1)		1.03	(0.90;1.18)	0.96	(0.81;1.14)
**Mother’s paid work**							
Yes	60.2	(58.1;62.3)		1.00		1.00	
No	60.8	(58.7;62.9)		0.97	(0.86;1.10)	0.90	(0.78;1.03)
**Child received vaccines exclusively in the public sector**							
Yes	60.2	(58.5;61.9)		1.00		1.00	
No	60.0	(56.3;63.7)		0.99	(0.84;1.18)	0.81	(0.65;1.01)
**Child received the recommended vaccines at 4 months old on the same date**							
Yes	76.9	(75.2;78.4)		1.00		1.00	
No	66.8	(64.0;69.5)		0.61	(0.52;0.71)	0.63	(0.53;0.75)
**Child with delayed vaccination in any vaccine up to 6 months old**							
Yes	56.7	(54.8;58.6)		0.27	(0.23;0.32)	0.28	(0.24;0.32)
No	82.7	(80.5;84.8)		1.00		1.00	

Vaccination coverage was lower among children from families in stratum A (OR = 0.74; 95%CI 0.58;0.93), children of mothers with lower level of education (OR = 0.75; 95%CI 0.61; 0.92), who did not receive the recommended vaccines at 4 months old on the same date (OR = 0.61; 95%CI 0.52;0.71) and those who experienced delays in any vaccines by 6 months old (OR = 0.27; 95%CI 0.23;0.32). Vaccination coverage was higher among children from families in consumption level C (OR = 1.26; 95%CI 1.10;1.44), with monthly income between BRL 3,001 and BRL 8,000 (OR = 1.37; 95%CI 1.14;1.64) and children of mothers aged 35 years or older (OR = 1.49; 95%CI 1.05;2.11). In the adjusted analysis, vaccination coverage remained lower among children in stratum A (OR = 0.68; 95%CI 0.53;0.87), children of mothers with lower level of education (OR = 0.70; 95%CI 0.54 ;0.90), who did not receive the vaccines at 4 months old on the same date (OR = 0.63; 95%CI 0.53;0.75) and who experienced delays in receiving any vaccines by six months old (OR = 0.28; 95%CI 0.24;0.32).

## DISCUSSION

Vaccination coverage with a complete schedule of doses administered was 30 percentage points lower than that proposed by the PNI, which is at least 90%.^3^ When taking into consideration valid doses, the coverage reached less than half and, considering timely doses, one-tenth had complete coverage. This scenario is concerning as it may lead to the accumulation of susceptible individuals, facilitating the spread of infectious agents that cause vaccine-preventable diseases and, consequently, hindering the achievement or maintenance of control and/or elimination of these diseases.

In another survey, conducted in all Brazilian capitals and in the Federal District in 2007, 83% of children had complete coverage in 2005,^10^ however, at that time, only seven vaccines (intradermal BCG, hepatitis B, tetravalent, oral polio, yellow fever, MMR and DTP) were part of the vaccination schedule, compared to the 13 currently included for children under 2 years of age. In a study conducted in Nepal in 2016, complete coverage was 78% (95%CI 74;81), with four vaccines included in the schedule.^11^ In the United States, 69.4% of children aged 18-35 months had complete coverage in a 2015-2020 cohort.^12^ Comparing vaccination coverage results with other studies is challenging, as most of them present their results for each vaccine individually rather than the set of vaccines. Another factor hindering comparisons with international studies is the different number of vaccines available in each country.

In this study, Sete Lagoas/Minas Gerais state was the municipality with the highest coverage with administered doses (79%), 2.3 times as high as Natal/Rio Grande do Norte (37%). In the United States, among children born in 2017-2018, the variation observed between states was 1.5 times, ranging the lowest coverage in Oklahoma (58%) to the highest in Massachusetts (85%).^14^ In England, coverage for most vaccines in 2021 was more homogeneous, remaining above 90% and below the 95% target in nearly all regions, with the exception of London, where coverage for all recommended vaccines was below 90%, reaching 82% for meningococcal B (part of the routine vaccination schedule in that country) and 83% for MMR.^13^

In this study, 6% of children did not receive any doses of vaccine, while in Mexico, in 2021, less than 1% received any dose,^15^ and in the United States, the percentage was similar in 2020.^12^

Delayed vaccination is a factor that impacts vaccination coverage. Children who did not experience delays until 6 months old had 31.5% higher coverage when compared to those who had at least one delay. Comparing delayed vaccination results with other studies available in the literature is also difficult, as they usually compare the delay for each vaccine individually rather than for the set of vaccines that should be administered within the same period. In India,^16^ a 23.1% delay in BCG and 29.3% for the first dose of DTP was observed, higher than the delays observed in the present study (6.1% and 11.5%, respectively).

In Argentina, in 2008,^17^ a 21.2% delay was observed for MMR, compared to 37.7% in this study; on the other hand, the delay for BCG was nearly twice as high, 12.1 %. In the Philippines, in 2016, in addition to a 25.6% delay for BCG, delays of 34.0% for the first dose and 50.7% for the second dose of the pneumococcal vaccine were observed,^18^ higher than those in this survey (5.9% and 10.3%, respectively).

In this study, the BCG vaccine showed the highest coverage, being the only one to meet the target recommended by the PNI (90%)^1^. In 2020, vaccination coverage for the Americas was 94% for BCG, 76% for HepB at birth, 85% for the third dose of IPV, 73% for the second dose of HRV; 84% for the third dose of the pneumococcal vaccine; 90% for the first dose and 72% for the second dose of MMR.^19^ Compared to this study, PAHO^19^ showed higher coverage for BCG, similar coverage for the first dose of MMR and third dose of the pneumococcal vaccine (first booster) and lower coverage for HepB at birth, third dose of IPV, second dose of HRV and second dose of MMR.

In a survey conducted in Canada in 2017 ^20^, coverage (complete scheme) for IPV (90.7%) was higher than that found in this study. Coverage for MMR was similar (90.2%), while coverage for pentavalent (75.8%), chickenpox (82.9%), meningococcal C (87.6%), pneumococcal (81.4%) and rotavirus (78.8%) were lower.

Complete coverage with administered doses was significantly different according to maternal education level, showing lower coverage among children of mothers with no formal education or with incomplete elementary education. The same finding was observed in Nepal,^15^ the Philippines and India.^21^

In stratum A, complete coverage with doses administered was less than 40% among children of mothers with lower level of education, which, when weighted, may have negatively impacted vaccination coverage in this stratum.

The results of this study should be considered in light of its limitations. Urban areas of the capitals, the Federal District and 12 inland municipalities were included. Although it is not possible to extrapolate the data to the whole country, the sample represents an important portion of the population. The interviewer’s access to families was hampered by urban insecurity, the COVID-19 pandemic and a lack of interest in participating, especially among families in higher socioeconomic strata. It is worth highlighting that the effect of these losses was minimized by calculating sampling weights.

Reading the digitized vaccination booklets was also challenging due to a lack of standardization in the way it was written down, as well as illegible date records and annotation errors. When the vaccination booklet was not presented during the interview and could not be retrieved, the children were considered unvaccinated, which may underestimate vaccination coverage. The magnitude and geographic scope of the sample, as well as the collection of vaccine administration dates directly from the vaccination booklet strengthen the robustness of the findings. 

Vaccination coverage is lower than expected and Brazil’s PNI requires action at all levels of government to recover the high levels achieved in past decades. Some actions should be at national level, while others should take into consideration the reality of each location. Identifying absentees and implementing an efficient active search strategies are essential to reduce vaccine delays and improve coverage. The simultaneous administration of vaccines in a single session reduces the number of visits and enables adherence to the schedule by the child´s guardian.

The data presented refer to the cohort of live births in 2017 and 2018 and may reflect a different reality from the current one for the same age group, as children born during the pandemic period may have different vaccination status and explanatory models. For this reason, periodic vaccination surveys are essential to provide measures to improve vaccination coverage.
