# Supplementary material for: Complete vaccination coverage of children born in 2017-2018, living in urban areas of state capitals and in 12 inland cities in Brazil: a population-based survey from a retrospective cohort study
Source: Epidemiol Serv Saude. 2024 Nov 1;33(spe2):e20231101. doi: 10.1590/S2237-96222024v33e20231101.especial2.en (PMC11537178; doi:10.1590/S2237-96222024v33e20231101.especial2.en)
Supplement: Translation

# Translation: Cobertura vacinal completa de crianças nascidas em 2017-2018, residentes nas áreas urbanas das capitais e em 12 cidades do interior do Brasil, inquérito de base populacional baseado em uma coorte retrospectiva

**DOI:** 10.1590/S2237-96222024v33e20231101.especial2.pt

**Authors:** José Cássio de Moraes, Ana Paula França, Ione Aquemi Guibu, Rita Barradas Barata, Carla Magda Allan Santos Domingues, Maria da Gloria Teixeira, Grupo ICV 2020, Adriana Ilha da Silva, Alberto Novaes Ramos, Ana Paula França, Andrea de Nazaré Marvão Oliveira, Antonio Fernando Boing, Carla Magda Allan Santos Domingues, Consuelo Silva de Oliveira, Ethel Leonor Noia Maciel, Ione Aquemi Guibu, Isabelle Ribeiro Barbosa Mirabal, Jaqueline Caracas Barbosa, Jaqueline Costa Lima, José Cássio de Moraes, Karin Regina Luhm, Karlla Antonieta Amorim Caetano, Luisa Helena de Oliveira Lima, Maria Bernadete de Cerqueira Antunes, Maria da Gloria Teixeira, Maria Denise de Castro Teixeira, Maria Fernanda de Sousa Oliveira Borges, Rejane Christine de Sousa Queiroz, Ricardo Queiroz Gurgel, Rita Barradas Barata, Roberta Nogueira Calandrini de Azevedo, Sandra Maria do Valle Leone de Oliveira, Sheila Araújo Teles, Silvana Granado Nogueira da Gama, Sotero Serrate Mengue, Taynãna César Simões, Valdir Nascimento, Wildo Navegantes de Araújo

**Affiliations:** 1 Santa Casa de São Paulo, Faculdade de Ciências Médicas, São Paulo, SP, Brasil; 2 Consultora independente, Brasília, DF, Brasil; 3 Universidade Federal da Bahia, Instituto de Saúde Coletiva, Salvador, BA, Brasil; Universidade Federal do Espírito Santo, Vitória, ES, Brasil; Universidade Federal do Ceará, Departamento de Saúde Comunitária, Fortaleza, CE, Brasil; Faculdade Ciências Médicas Santa Casa de São Paulo, São Paulo, SP, Brasil; Secretaria de Estado da Saúde do Amapá, Macapá, AP, Brasil; Universidade Federal de Santa Catarina, SC, Brasil; Organização Pan-Americana da Saúde, Brasília, DF, Brasil; Instituto Evandro Chagas, Belém, PA, Brasil; Faculdade de Ciências Médicas Santa Casa de São Paulo, Departamento de Saúde Coletiva, São Paulo, SP, Brasil; Universidade Federal do Rio Grande do Norte, Natal, RN, Brasil; Universidade Federal do Ceará, Departamento de Saúde Comunitária, Fortaleza, CE, Brasil; Universidade Federal de Mato Grosso, Cuiabá, MT, Brasil; Universidade Federal do Paraná, Curitiba, PR, Brasil; Universidade Federal de Goiás, Goiânia, GO, Brasil; Universidade Federal do Piauí, Teresina, PI, Brasil; Universidade de Pernambuco, Faculdade de Ciências Médicas, Pernambuco, PE, Brasil; Instituto de Saúde Coletiva, Universidade Federal da Bahia, Salvador, BA, Brasil; Secretaria de Estado da Saúde de Alagoas, Maceió, AL, Brasil; Universidade Federal do Acre, Rio Branco, AC, Brasil; Universidade Federal do Maranhão, Departamento de Saúde Pública, São Luís, MA, Brasil; Universidade Federal de Sergipe, Aracaju, SE, Brasil; Secretaria Municipal de Saúde, Boa Vista, RR, Brasil; Fundação Oswaldo Cruz, Mato Grosso do Sul, Campo Grande, MS, Brasil; Fundação Oswaldo Cruz, Escola Nacional de Saúde Pública Sergio Arouca, Rio de Janeiro, RJ, Brasil; Universidade Federal do Rio Grande do Sul, Porto Alegre, RS, Brasil; Fundação Oswaldo Cruz, Instituto de Pesquisa René Rachou, Belo Horizonte, MG, Brasil; Secretaria de Desenvolvimento Ambiental de Rondônia, Porto Velho, RO, Brasil; Universidade de Brasília, Brasília, DF, Brasil

**Keywords:** Cobertura Vacinal, Inquéritos Epidemiológicos, Programas de Imunização, Atraso Vacinal, Vacinação

## Abstract

**Objetivo:**

Estimar a cobertura vacinal em crianças nascidas entre 2017-2018, residentes nas áreas urbanas das capitais, do Distrito Federal e em 12 municípios do interior do Brasil, e identificar fatores associados.

**Métodos:**

Inquérito domiciliar realizado entre 2020-2022, em crianças até 24 meses. Estimou-se a cobertura vacinal segundo características da família, da mãe e da criança.

**Resultados:**

Nas 37.801 crianças da amostra, a cobertura completa (doses aplicadas) foi de 60,1% (IC_95_% 58,6;61,6), e 6,1% (IC_95_% 5,4;7,0) não receberam qualquer vacina. A cobertura foi menor em crianças de mães com menor instrução (OR = 0,70; IC_95_% 0,54;0,90) e nas que tinham atraso em qualquer vacina até os 6 meses de vida (OR = 0,28; IC_95_% 0,24;0,32).

**Conclusão:**

As coberturas vacinais estão aquém do esperado. É necessário adotar estratégias de comunicação efetivas para reforçar a importância da vacinação de rotina, prevenindo atrasos e abandono do esquema vacinal, para retomar os altos níveis alcançados em décadas passadas.

## Contribuições do estudo

**Principais resultados:**

A cobertura vacinal com esquema completo sem febre amarela foi 60%, e menor em mães com baixa escolaridade e em crianças com atraso vacinal. Com exceção da vacina BCG intradérmica, as demais não atingiram os níveis recomendados.

**Implicações para os serviços:**

O processo de vacinação nas unidades básicas de saúde pode ser melhorado, propiciando a aplicação simultânea de doses previstas em uma mesma sessão e busca ativa de faltosos, a fim de se reduzir o atraso vacinal e facilitar o acesso.

**Perspectivas:**

A discussão dos resultados nos três níveis de gestão poderá subsidiar propostas para aprimorar o funcionamento dos serviços de saúde, principalmente do processo de trabalho das salas de vacina, com o objetivo de melhoria da cobertura vacinal.

## INTRODUÇÃO

Conhecer a real cobertura vacinal de uma comunidade é fundamental para propor ações que melhorem ou mantenham o controle de doenças imunopreveníveis.^1^ Nas primeiras duas décadas após a implantação do Programa Nacional de Imunizações (PNI), em 1973, as coberturas vacinais foram baixas, mas, progressivamente, atingiram níveis ideais para controlar, eliminar ou erradicar algumas doenças imunopreveníveis.^2^ A partir de 2016, no entanto, constatou-se queda da cobertura para todas as vacinas disponíveis no PNI, que se acentuou nos anos pandêmicos de covid-19.^3,4^

A ocorrência de epidemias, mesmo com estimativas de coberturas altas, demonstra a imprecisão dessas estimativas, evidenciada por inquéritos domiciliares. A heterogeneidade de cobertura, nem sempre corretamente identificada, representa risco de acúmulo de suscetíveis, com potencial para introdução e manutenção da circulação de agentes infecciosos. A epidemia de sarampo, que em 1997 surpreendeu o programa de vigilância epidemiológica, alertou para a importância de se conhecerem corretamente as coberturas vacinais.^5^

No Brasil, a cobertura vacinal é monitorada com estimativas calculadas com dados gerados pelo Sistema de Informação do PNI (SI-PNI). A precisão dessas estimativas é impactada por problemas operacionais que afetam a qualidade e completude dos dados, tais como a subnotificação dos serviços privados de vacinação, os sistemas locais, que não se integram ao SI-PNI, e as deficiências na estrutura tecnológica e nos recursos humanos para alimentação do sistema nas unidades de saúde.^2^

Outro fator que contribui para a imprecisão do registro de dados no SI-PNI é a quantidade de vacinas presentes no calendário atual: 13 vacinas e 23 doses até os 24 meses. Essas vacinas são aplicadas em mais de 36 mil unidades do Sistema Único de Saúde (SUS) em todos os municípios brasileiros, por meio de várias estratégias, tais como rotina; cobertura/bloqueio de foco; campanhas indiscriminadas ou seletivas; vacinação casa a casa e em escolas, entre outras.^3^

No inquérito de cobertura vacinal realizado em 2007 em todas as capitais brasileiras, em crianças de 18 a 30 meses, a cobertura completa com doses aplicadas ultrapassou 80%.^5^ Realizar inquéritos periodicamente não apenas permite estimar esses indicadores de forma mais precisa, mas também avaliar as tendências e identificar aspectos das condições de vida que impactam o processo de vacinação.^6^

O presente inquérito teve como objetivo principal estimar a cobertura vacinal em crianças nascidas em 2017-2018, residentes nas áreas urbanas das capitais, do Distrito Federal e em 12 municípios do interior do Brasil e identificar fatores associados à vacinação.

## MÉTODOS

Trata-se de inquérito de base populacional baseado em coorte retrospectiva, realizada de 2020 a 2022, que verificou o cumprimento do calendário vacinal do PNI e os fatores associados à vacinação.^7^

A população de estudo foi composta por crianças nascidas em 2017 e 2018, residentes nas áreas urbanas das 26 capitais das Unidades Federativas (UF), do Distrito Federal e nos seguintes municípios: Imperatriz/MA, Sobral/CE, Caruaru/PE e Vitória da Conquista/BA (Nordeste); Sete Lagoas/MG, Petrópolis/RJ e Campinas/SP (Sudeste); Londrina/PR, Joinville/SC e Rio Grande/RS (Sul); Rondonópolis/MT e Rio Verde/GO (Centro-Oeste). Os municípios do interior elegíveis tinham população acima de 100 mil habitantes em 2020, se localizaram fora da região metropolitana de sua respectiva capital e foram escolhidos por conveniência.^7^

Na primeira etapa, classificaram-se os setores censitários em cada um dos 39 municípios em 4 estratos socioeconômicos (A, B, C e D – sendo A o de melhor e D o de pior condição socioeconômica). Em cada município, os pontos de corte da estratificação foram diferentes, considerando a renda nominal do chefe de família, e percentual de chefes de família com renda superior a 20 salários mínimos e o percentual de chefes de família alfabetizados, com os dados do censo demográfico de 2010.^7^

O conjunto dos municípios estudados foi dividido em 88 domínios de interesse (inquéritos), variando de 1 a 4 por município, dependendo do número de nascidos vivos registrados no Sistema de Informação sobre Nascidos Vivos em 2017 e 2018. O cálculo do tamanho da amostra baseou-se em cobertura vacinal de 70%, efeito de desenho de 1,4 e nível de confiança de 95%, resultando em 452 em cada área de interesse, totalizando um tamanho da amostra prevista de 39.776 crianças.^7^ A amostragem foi por conglomerados de forma sistemática. 

O questionário completo da pesquisa foi composto por nove blocos, porém, para este artigo, foram analisadas variáveis relacionadas às características da família (estrato socioeconômico, nível de consumo familiar, renda mensal familiar e programa de transferência de renda), da mãe da criança (escolaridade e faixa etária), da criança (serviço no qual recebeu a vacina) e do processo de vacinação (coincidência de datas das vacinas recomendadas aos 4 meses e atraso vacinal em qualquer vacina prevista até os 6 meses). O nível de consumo das famílias foi definido segundo critério da Associação Brasileira de Empresas de Pesquisa.^8^

Para o cálculo das coberturas vacinais, foi obtido o registro fotográfico da caderneta de vacinação; para a criança que não tinha caderneta disponível, buscou-se a ficha espelho no SI-PNI, e, quando não encontrada no sistema, ela foi considerada não vacinada.^9^

Foi realizada a junção de diferentes vacinas voltadas para a prevenção das mesmas doenças (por exemplo, vacina tríplice bacteriana [difteria, tétano e pertússis, DTP], tetravalente, pentavalente ou hexavalente para as respectivas doenças), a fim de se calcular corretamente as coberturas, considerando-se as vacinas aplicadas pelo setor público e por serviços privados.^9^

A diferença entre as datas registradas na caderneta e a data de nascimento da criança permitiu classificar as doses aplicadas em válidas e/ou oportunas. Doses válidas foram aquelas aplicadas desde 15 dias antes da data aprazada pelo PNI, respeitando o intervalo mínimo recomendado para cada dose. Doses oportunas foram as aplicadas entre 15 dias antes e até 30 dias após a data prevista.^3,7^

O indicador de cobertura vacinal completa (até 24 meses) foi definido considerando-se a aplicação do conjunto das doses e os reforços das vacinas previstas no calendário oficial do PNI: BCG; hepatite B (hepB); pentavalente (penta: DTP + *hemophilus influenza* B + hepatite B) – primeira + segunda + terceira doses; poliomielite 1, 2 e 3 inativada (VIP: primeira + segunda + terceira doses); rotavírus humano (VRH: primeira + segunda doses); meningocócica C (menC: primeira + segunda doses + reforço); pneumocócica 10-valente (pneumo-10: primeira + segunda doses); sarampo, caxumba e rubéola (tríplice viral: primeira + segunda doses + reforço); hepatite A (hepA: primeira dose); varicela (VZ: dose única); poliomielite 1 e 3 atenuada (VOPb: reforço); difteria, tétano e pertússis (DTP: reforço). A vacina febre amarela (FA) foi excluída do cálculo, pois não fazia parte do calendário de rotina em alguns municípios.^3,7^ Para o cálculo da taxa de abandono para vacinas com múltiplas doses, utilizou-se a fórmula:


$$\left(\frac{\text{número de crianças que não completaram o esquema}}{\text{número de crianças que tomaram a primeira dose}}\right)\times 100.$$

O atraso vacinal foi caracterizado quando a dose foi aplicada após 30 dias da data recomendada no calendário do PNI.^7^

A cascata da cobertura para cada vacina foi calculada com o número de vacinados no numerador e, no denominador, o número de crianças que receberam a dose da vacina imediatamente anterior. Assim, para calcular a cobertura da vacina hepB ao nascer, o denominador foi o número de crianças que tinham recebido a vacina BCG.

Para identificar as diferenças nas coberturas vacinais entre os municípios, a precisão da estimativa pontual foi feita pelos intervalos de confiança de 95% (IC_95_%). Para analisar fatores associados às coberturas vacinais, foram estimadas as razões de chance *(odds rat*io, OR) por meio de regressão logística. As associações significativas entre as variáveis e a cobertura vacinal completa, na análise bruta, foram ajustadas por renda familiar, escolaridade e idade da mãe. Todas as análises foram realizadas no programa Stata, versão 17, utilizando o módul*o surv*ey, considerando-se os pesos amostrais e o delineamento do estudo.

O estudo foi aprovado pelos Comitês de Ética em Pesquisa com Seres Humanos do Instituto de Saúde Coletiva da Universidade Federal da Bahia, sob parecer nº 3.366.818, em 4 de junho de 2019, com Certificado de Apresentação de Apreciação Ética (CAAE) 4306919.5.0000.5030, e da Irmandade da Santa Casa de São Paulo, sob parecer nº 4.380.019, em 4 de novembro de 2020, com CAAE 39412020.0.0000.5479. 

## RESULTADOS

Foram realizadas 37.801 entrevistas (95,0%) nos 39 municípios. A maior taxa de perda ocorreu no estrato socioeconômico A (16,2%), e não houve perda nos estratos C e D (tabela não apresentada).

A vacina com maior cobertura (doses aplicadas) foi a BCG, 89,8% (IC_95_% 88,6;90,8), e as menores foram segunda dose de VRH, primeiro reforço de menC e segunda dose de tríplice viral, 82,2% (IC_95_% 80,9;83,5). Considerando-se doses válidas, o primeiro reforço da pneumo-10 teve a menor cobertura: 73,8% (IC_95_% 72,0;75,4) (Tabela 1). 

**Tabela 1:** Cobertura vacinal das vacinas previstas no calendário do Programa Nacional de Imunizações (PNI) de acordo com a classificação de doses (aplicadas, válidas ou oportunas) e razão entre as coberturas. Capitais, Distrito Federal e 12 municípios do interior do Brasil. Inquérito nacional de cobertura vacinal, 2020 (n = 37.801)

**Vacina**	**Cobertura completa aos 24 meses**							
	**Doses aplicadas**		**Doses válidas**		**Doses oportunas**		**Válidas/ aplicadas**	**Oportunas/ aplicadas**
	**%**	**(IC**_95_ **% )**	**%**	**(IC**_95_ **% )**	**%**	**(IC**_95_ **% )**		
BCG	89,8	(88,6;90,8)	89,8	(88,6;90,8)	83,6	(82,3;84,8)	1,00	0,93
Hepatite B (HepB)	88,8	(87,6;89,9)	88,8	(87,5;89,9)	85,7	(84,5;86,8)	1,00	0,97
Difteria, tétano, pertússis, hepatite B e *Haemophilus influenzae B* (penta - 3ª dose)	88,1	(86,9;89,2)	87,5	(86,3;88,6)	56,3	(54,6;58,1)	0,99	0,64
Poliomielite 1, 2 e 3 inativada (VIP - 3ª dose)	88,0	(86,8;89,1)	87,5	(86,3;88,6)	58,4	(56,6;60,0)	0,99	0,66
Pneumocócica 10-valente (Pneumo-10 - 2ª dose)	90,4	(89,4;91,4)	89,9	(88,8;90,9)	79,9	(78,5;81,1)	0,82	0,88
Rotavírus humano (VRH - 2ª dose)	82,2	([80,9;83,4)	75,8	(74,3;77,2)	66,2	(64,6;67,8)	0,92	0,81
Meningocócica C (menC -2ª dose)	89,5	(88,4;90,4)	88,7	(87,6;89,7)	61,8	(60,1;63,4)	0,99	0,69
Hepatite A (hepA)	88,2	(87,1;89,3)	87,0	(85,8;88,1)	49,9	(48,4;51,3)	0,99	0,57
Sarampo, caxumba e rubéola (tríplice viral - 1ª dose)	90,9	(89,9;91,9)	90,0	(88,9;91,0)	52,3	(50,6;53,9)	0,99	0,58
Sarampo, caxumba e rubéola (tríplice viral - 2ª dose)	82,2	(80,9;83,5)	81,1	(79,8;82,3)	40,2	(38,8;41,7)	0,99	0,49
Varicela (VZ)	87,1	(85,9;88,1)	79,2	(77,6;80,7)	46,5	(45,0;48,0)	0,91	0,53
Pneumocócica 10-valente (pneumo-10) 1º reforço	85,0	(83,8;86,2)	73,8	(72,0;75,4)	43,9	(42,2;45,5)	0,87	0,52
Meningocócica C (menC - 1º reforço)	82,2	(81,0;83,4)	76,6	(75,2;78,0)	42,0	(40,4;43,6)	0,93	0,51
Difteria, tétano, pertússis (DTP - 1º reforço)	84,2	(83,1;85,3)	83,5	(82,3;84,6)	36,4	(34,9;38,0)	0,99	0,43
Poliomielite 1 e 3 atenuada (VOPb - 1º reforço)	86,2	(85,1;87,3)	81,8	(80,4;83,0)	43,1	(41,6;44,6)	0,95	0,50

As coberturas com o esquema completo (doses aplicadas) das vacinas para doenças objeto de política de erradicação (poliomielite) e de eliminação regional (sarampo e rubéola) foram, respectivamente, de 88,0% (IC_95_% 86,8;89,1) e 82,2% (IC_95_% 80,9;83,5), inferiores ao proposto pelo plano de erradicação/eliminação, que é de 95% (Tabela 1).

A maior diferença relativa entre doses aplicadas e válidas foi observada na segunda dose da pneumo-10. Todas as vacinas aplicadas no segundo ano de vida apresentaram cobertura vacinal abaixo de 50% com doses oportunas (Tabela 1).

A Figura 1 mostra a evolução das coberturas de acordo com a sequência prevista no calendário vacinal. Entre os que receberam a vacina BCG (89,6%), somente 60,1% completaram o esquema, representando queda importante (32,9%). No estrato socioeconômico A, a cobertura da BCG foi de 83,1% (IC_95_% 78,6;87,2) e, no final do seguimento, somente 53,4% (IC_95_% 48,2;58,6) das crianças receberam todas as vacinas. No estrato D, 90,7% (IC_95_% 89,2;92,3) das crianças receberam a BCG e a cascata terminou com 60,8% (IC_95_% 58,7;62,8). As quedas mais evidentes foram observadas no momento da aplicação da segunda dose da VRH e segunda dose da tríplice viral.

**Gráfico 1:** Cascata de doses aplicadas das vacinas previstas no calendário do PNI, segundo estrato socioeconômico. Capitais, Distrito Federal e 12 municípios do interior do Brasil. Inquérito nacional de cobertura vacinal, 2020 (n = 37.801)

No conjunto dos municípios, as taxas de abandono (doses aplicadas) da penta foi 4,0% e da VIP foi 1,4%. Para as vacinas VRH, pneumo-10 e menC, as taxas foram de 6,5%, 2,2% e 2,8%, respectivamente. Para o esquema da tríplice viral, a taxa de abandono foi de 9,6% (Figura 1). 

A cobertura completa para o conjunto dos municípios estudados (capitais + interior) foi de 60,1% (IC_95_% 58,6;61,6) com doses aplicadas e 43,5% (IC_95_% 41,9;45,2) com doses válidas, representando uma diferença de 27,6% (Tabela 2). A cobertura completa com doses oportunas foi muito baixa, 9,8% (IC_95_% 8,8;10,9), com diferença de 83,7% em relação às doses aplicadas. O percentual de crianças que não recebeu nenhuma dose de vacina foi de 6,1% (IC_95_% 5,3;6,9). 

**Tabela 2:** Cobertura vacinal completa até os 24 meses segundo município e classificação de doses (aplicadas, válidas e oportunas). Capitais, Distrito Federal e 12 municípios do interior do Brasil. Inquérito nacional de cobertura vacinal, 2020 (n = 37.801)

Município	Cobertura completa aos 24 meses						**Válidas/aplicadas**	**Oportunas/aplicadas**
	**Doses aplicadas**		**Doses válidas**		**Doses oportunas**			
	**%**	**(IC**_95_ **% )**	**%**	**(IC**_95_ **% )**	**%**	**(IC**_95_ **% )**		
Porto Velho	64,8	(57,9;71,2)	54,0	(46,7;61,1)	8,9	(5,4;14,4)	0,83	0,14
Rio Branco	60,8	(53,2;67,8)	43,3	(38,0;48,6)	6,1	(2,6;13,6)	0,71	0,10
Manaus	54,1	(49,6;58,1)	42,9	(39,0;46,9)	3,2	(2,0;4,8)	0,79	0,06
Boa Vista	60,0	(48,7;70,3)	51,8	(39,6;63,8)	9,7	(6,0;15,4)	0,86	0,16
Belém	57,5	(47,4;67,0)	34,7	(24,9;46,0)	4,8	(2,0;10,8)	0,60	0,08
Macapá	35,8	(28,1;44,4)	25,8	(17,8;35,8)	4,2	(2,1;8,1)	0,72	0,12
Palmas	67,5	(60,5;73,8)	59,3	(51,5;66,2)	5,6	(2,9;10,5)	0,88	0,08
Imperatriz	46,3	(40,9;51,7)	34,2	(28,7;40,1)	4,1	(2,1;6,1)	0,74	0,09
São Luís	51,6	(43,0;60,1)	36,2	(27,4;46,1)	2,8	(1,4;5,1)	0,70	0,05
Teresina	73,7	(63,0;82,1)	64,6	(56,3;72,2)	10,2	(6,0;16,6)	0,88	0,14
Fortaleza	54,0	(47,3;60,6)	45,3	(39,1;51,7)	11,1	(8,0;15,2)	0,84	0,21
Sobral	57,7	(47,2;67,5)	47,1	(37,5;57,0)	17,4	(11,0;26,5)	0,82	0,30
Natal	36,6	(26,7;47,3)	31,5	(22,8;41,6)	5,3	(2,1;12,5)	0,86	0,14
João Pessoa	42,6	(36,3;49,2)	33,4	(27,2;40,2)	4,6	(3,3;6,4)	0,78	0,11
Caruaru	68,8	(60,1;76,3)	57,7	(49,4;65,7)	11,9	(7,8;17,9)	0,84	0,17
Recife	56,9	(49,6;63,9)	42,4	(34,9;50,2)	8,0	(5,4;11,9)	0,75	0,14
Maceió	58,3	(50,2;66,0)	42,2	(35,2;49,5)	7,5	(4,4;12,5)	0,72	0,13
Aracaju	65,3	(58,6;71,4)	52,4	(47,4;57,3)	15,1	(11,6;19,5)	0,80	0,23
Salvador	64,9	(60,5;69,1)	52,6	(47,1;58,1)	14,8	(9,5;22,3)	0,81	0,23
Vitória da Conquista	61,7	(51,2;71,1)	54,9	(43,5;65,9)	11,8	(5,7;22,9)	0,89	0,19
Belo Horizonte	63,8	(59,5;67,9)	46,2	(40,2;52,3)	13,2	(9,9;17,4)	0,72	0,21
Sete Lagoas	79,1	(75,1;82,7)	61,6	(53,6;69,1)	19,5	(15,2;24,6)	0,78	0,25
Vitória	57,1	(50,6;63,4)	34,3	(22,9;47,7)	6,7	(4,2;10,9)	0,60	0,12
Petrópolis	69,4	(61,0;76,8)	50,3	(34,4;66,1)	7,2	(4,3;11,7)	0,72	0,10
Rio de Janeiro	51,7	(45,8;57,4)	34,6	(29,9;39,7)	11,9	(8,9;15,7)	0,67	0,23
Campinas	63,0	(54,2;71,1)	43,3	(35,8;51,1)	11,1	(8,1;15,0)	0,69	0,18
São Paulo	64,0	(60,1;67,7)	44,2	(39,2;49,4)	13,1	(10,2;16,7)	0,69	0,20
Curitiba	74,4	(66,3;81,1)	49,2	(39,8;58,7)	6,6	(3,2;12,6)	0,66	0,09
Londrina	66,6	(50,5;79,6)	57,7	(41,9;72,1)	17,6	(8,7;32,3)	0,87	0,26
Joinville	71,4	(65,3;76,9)	58,4	(52,5;64,0)	11,4	(7,6;16,5)	0,82	0,16
Florianópolis	49,6	(40,8;58,5)	25,5	(19,0;33,4)	4,0	(2,1;7,5)	0,51	0,08
Porto Alegre	65,2	(59,7;70,3)	48,4	(42,8;54,0)	7,9	(5,4;11,5)	0,74	0,12
Rio Grande	57,2	(47,7;66,2)	37,8	(20,7;58,5)	11,3	(5,6;21,2)	0,66	0,20
Campo Grande	54,2	(48,2;60,1)	40,9	(35,9;46,2)	5,3	(3,7;7,6)	0,75	0,10
Cuiabá	60,9	(53,2;68,1)	46,8	(40,1;53,6)	5,3	(2,5;11,0)	0,77	0,09
Rondonópolis	44,9	(36,1;54,1)	36,9	(28,4;46,3)	8,5	(5,1;14,0)	0,82	0,19
Goiânia	56,7	(50,2;62,9)	47,9	(41,5;54,4)	10,6	(6,1;17,9)	0,84	0,19
Rio Verde	38,5	(30,4;47,3)	28,7	(23,5;34,5)	5,2	(2,3;11,5)	0,75	0,14
Brasília	73,1	(69,3;76,6)	55,2	(50,5;59,8)	15,8	(12,8;19,5)	0,76	0,22
Capitais (n = 31.001)	59,9	(58,3;61,5)	43,1	(41,3;44,9)	9,7	(8,6;10,9)	0,72	0,16
Interior (n = 6.800)	62,3	(58,9;65,5)	48,0	(44,3;51,7)	11,0	(9,1;13;3)	0,77	0,18
Total (n = 37.801)	60,1	(58,6;61,6)	43,5	(41,9;45,2)	9,8	(8,8;10,9)	0,72	0,16

As coberturas completas com doses aplicadas, válidas ou oportunas foi heterogênea nos 39 municípios estudados (Tabela 2). Sete Lagoas/MG teve as melhores coberturas completas com doses aplicadas, válidas e oportunas. As menores coberturas com doses aplicadas foram constatadas em Natal/RN, com doses válidas em Rio Verde/GO e, com doses oportunas, em Manaus/AM. As maiores diferenças de coberturas com doses válidas e aplicadas (40,0%) foram observadas nos municípios de Belém/PA e Vitória/ES. Considerando-se doses oportunas, a maior diferença na cobertura vacinal foi encontrada em São Luís/MA (95,0%). 

Cinco municípios tiveram coberturas completas acima de 70% com doses aplicadas (Teresina/PI, Sete Lagoas/MG, Curitiba/PR, Joinville/SC e Brasília/DF), enquanto em sete foram menores que 50% (Macapá/AP, Imperatriz/MA, Natal/RN, João Pessoa/PB, Florianópolis/SC, Rondonópolis/MT e Rio Verde/GO). Considerando-se somente doses válidas, apenas dois municípios (Teresina/PI e Sete Lagoas/MG) tiveram cobertura maior que 60%. Com doses oportunas, nenhum município teve cobertura superior a 20%. Na região Norte, o município com maiores coberturas (doses aplicadas e válidas) foi Palmas/TO; no Nordeste, Teresina/PI; no Sudeste, Sete Lagoas/MG; no Sul, Joinville/SC; e, no Centro-Oeste, Brasília/DF (Tabela 2).

O número médio de visitas às salas de vacinação para o cumprimento do calendário foi de 10,8, três a mais que o proposto pelo PNI, e 95% das crianças que completaram o esquema necessitaram de até 14 visitas (dados não apresentados em tabela).

O atraso na aplicação das vacinas ao nascer foi de 6,4% (IC_95_% 5,6;7,3). Aos 4 meses, quando são indicadas quatro vacinas, 21,5% (IC_95_% 20,2;22,8) das crianças tinham atraso em pelo menos uma. Nos primeiros 6 meses de vida, 51,4% (IC_95_% 49,6;53,3) tiveram atraso em qualquer uma. Na primeira dose da tríplice viral, que deve ser aplicada aos 12 meses, o atraso foi de 45,9% (IC_95_% 44,3;47,5) (dados não apresentados em tabela). 

A Tabela 3 mostra a cobertura completa segundo presença de atraso vacinal. A cobertura foi menor para as crianças que tiveram atraso em qualquer uma das vacinas até os 6 meses de vida: 56,7% (IC_95_% 54,8;58,6), em relação às que não tiveram atraso: 82,7% (IC_95_% 80,5;84,8). O atraso na aplicação das vacinas ao nascer acarretou diferença relativa de 17,4% na cobertura completa. Diferença semelhante foi observada quando o atraso ocorreu em qualquer das vacinas aplicadas aos 4 meses. Qualquer atraso até os 6 meses de vida resultou em diferença de 31,4% na cobertura completa. 

**Tabela 3:** Cobertura vacinal completa até os 24 meses de doses aplicadas segundo a ocorrência de atraso em pelo menos uma vacina de acordo com o período previsto para aplicação das vacinas. Capitais, Distrito Federal e 12 municípios do interior do Brasil. Inquérito nacional de cobertura vacinal, 2020 (n = 37.801)

Período previsto no calendário para aplicação	**Cobertura vacinal completa**				
	**Atraso vacinal**				**Diferença atribuída ao atraso** ^a^ **%**
	**Não**		**Sim**		
	**%**	**(IC**_95_ **% )**	**%**	**(IC**_95_ **% )**	
Ao nascer	69,0	(67,5;70,4)	57,0	(50,9;62,8)	17,4
Aos 2 meses	72,3	(70,8;73,7)	48,6	(44,3;53,0)	32,8
Aos 3 meses	70,4	(68,9;71,9)	41,8	(38,6;45,1)	40,6
Aos 4 meses	77,4	(75,8;78,9)	64,5	(61,7;67,2)	16,7
Aos 5 meses	73,3	(71,6;75,0)	53,4	(51,1;55,7)	27,1
Aos 6 meses	75,2	(73,3,76,9)	59,9	(57,6;62,2)	20,3
Até os 6 meses	82,7	(80,5;84,8)	56,7	(54,8;58,6)	31,4

a)[(cobertura sem atraso – cobertura com atraso) / cobertura sem atraso x 100].

A cobertura vacinal (doses aplicadas) não foi diferente quando a criança recebeu todas as vacinas exclusivamente no setor público ou se a aplicação de ao menos uma foi feita no setor privado: 60,2% (IC_95_% 58,5;61,9) e 60,0% (IC_95_% 56,3;63,7), respectivamente. 

**Tabela 4:** Cobertura vacinal completa de doses aplicadas até os 24 meses e razões de chance ( *odds ratio* , OR) brutas e ajustadas segundo características das famílias, das mães e das crianças. Capitais, Distrito Federal e 12 municípios do interior do Brasil. Inquérito nacional de cobertura vacinal, 2020 (n = 37.801)

Variáveis				**Análise bruta**		**Análise ajustada**	
	**%**	**(IC**_95_ **% )**		**OR**	**(IC**_95_ **% )**	**OR**	**(IC**_95_ **% )**
**Estrato socioeconômico**							
A	53,4	(48,2;58,6)		0,74	(0,58;0,93)	0,68	(0,53;0,87)
B	59,4	(54,9;63,8)		0,95	(0,77;1,16)	0,86	(0,69;1,07)
C	62,4	(60,0;64,8)		1,07	(0,94;1,23)	1,02	(0,88;1,19)
D	60,8	(58,7;62,8)		1,00		1,00	
**Nível de consumo familiar**							
A	60,3	(51,9;68,0)		1,10	(0,78;1,56)	0,88	(0,55;1,42)
B	60,8	(57,1;64,3)		1,13	(0,95;1,34)	1,05	(0,81;1,35)
C	63,4	(60,9;65,8)		1,26	(1,10;1,44)	1,16	(1,00;1,36)
D	57,8	(55,7;59,9)		1,00		1,00	
**Renda mensal familiar (R$)**							
≤ 1.000	58,1	(55,3;60,8)		1,00		1,00	
1.001 a 3.000	61,6	(59,2;64,0)		1,16	(1,00;1,35)	1,09	(0,93;1,29)
3.001 a 8.000	65,5	(62,1;68,7)		1,37	(1,14;1,64)	1,17	(0,95;1,44)
≥ 8.001	59,9	(54,2;65,4)		1,08	(0,83;1,40)	0,88	(0,66;1,18)
**Escolaridade da mãe**							
Sem instrução/fundamental incompleto	53,5	(49,5;57,3)		0,75	(0,61;0,92)	0,70	(0,54;0,90)
Fundamental completo	58,9	(55,0;62,6)		0,93	(0,76;1,14)	0,90	(0,70;1,17)
Médio completo	62,4	(60,3;64,5)		1,08	(0,93;1,26)	0,98	(0,82;1,18)
Superior completo	60,6	(57,5;63,6)		1,00		1,00	
**Faixa etária da mãe**							
< 20 anos	51,6	(43,3;59,9)		1,00		1,00	
20 a 34 anos	59,5	(57,5;61,4)		1,37	(0,97;1,95)	1,26	(0,82;1,92)
35 anos ou mais	61,4	(58,9;63,8)		1,49	(1,05;2,11)	1,29	(0,78;2,14)
**Programa de transferência de renda**							
Sim	59,5	(56,9;62,0)		1,00		1,00	
Não	60,3	(58,4;62,1)		1,03	(0,90;1,18)	0,96	(0,81;1,14)
**Trabalho remunerado da mãe**							
Sim	60,2	(58,1;62,3)		1,00		1,00	
Não	60,8	(58,7;62,9)		0,97	(0,86;1,10)	0,90	(0,78;1,03)
**Criança vacinada exclusivamente no serviço público**							
Sim	60,2	(58,5;61,9)		1,00		1,00	
Não	60,0	(56,3;63,7)		0,99	(0,84;1,18)	0,81	(0,65;1,01)
**Criança recebeu na mesma data as vacinas recomendadas aos 4 meses de vida**							
Sim	76,9	(75,2;78,4)		1,00		1,00	
Não	66,8	(64,0;69,5)		0,61	(0,52;0,71)	0,63	(0,53;0,75)
**Criança com atraso vacinal em qualquer vacina até os 6 meses de vida**							
Sim	56,7	(54,8;58,6)		0,27	(0,23;0,32)	0,28	(0,24;0,32)
Não	82,7	(80,5;84,8)		1,00		1,00	

As coberturas vacinais foram menores nas crianças de famílias do estrato A (OR = 0,74; IC_95_% 0,58;0,93), filhas de mães menos escolarizadas (OR = 0,75; IC_95_% 0,61;0,92), que não receberam na mesma data as vacinas recomendadas aos quatro meses de vida (OR = 0,61; IC_95_% 0,52;0,71) e que tiveram atraso em qualquer vacina até os 6 meses de vida (OR = 0,27; IC_95_% 0,23;0,32). As coberturas vacinais foram maiores nas crianças de famílias de nível de consumo C (OR = 1,26; IC_95_% 1,10;1,44), com renda mensal entre R$ 3.001 e R$ 8.000 (OR = 1,37; IC_95_% 1,14;1,64) e filhas de mães que tinham 35 anos ou mais (OR = 1,49; IC_95_% 1,05;2,11). Na análise ajustada permaneceram menores as coberturas vacinais nas crianças do estrato A (OR = 0,68; IC_95_% 0,53;0,87), filhas de mães menos escolarizadas (OR = 0,70; IC_95_% 0,54;0,90), que não receberam na mesma data as vacinas aos 4 meses de vida (OR = 0,63;IC_95_% 0,53;0,75) e que tiveram atraso em qualquer vacina até os 6 meses de vida (OR = 0,28; IC_95_% 0,24;0,32).

## DISCUSSÃO

A cobertura vacinal com esquema completo de doses aplicadas foi 30 pontos percentuais inferior ao proposto pelo PNI, que é de pelo menos 90%.^3^ Ao se considerarem doses válidas, alcançou menos da metade e, considerando -se doses oportunas, um décimo tinham cobertura completa. Esse cenário é preocupante, por possibilitar acúmulo de suscetíveis, facilitando a disseminação dos agentes infecciosos causadores das doenças evitáveis por vacinação e, consequentemente, dificultando alcançar ou manter o controle e/ou a eliminação dessas doenças.

Em outro inquérito, realizado em todas as capitais brasileiras e no Distrito Federal em 2007, 83% das crianças tinham cobertura completa em 2005,^10^ porém, naquela ocasião, apenas sete vacinas (BCG intradérmica, hepatite B, tetravalente, poliomielite oral, febre amarela, tríplice viral e DTP) faziam parte do calendário vacinal, em vez das 13 que estão incluídas no atual calendário para crianças menores de 2 anos de idade. Em um estudo realizado no Nepal, em 2016, a cobertura completa foi de 78% (IC_95_% 74;81), com quatro vacinas compondo o calendário.^11^ Nos Estados Unidos, 69,4% de crianças de 18-35 meses tinham cobertura completa numa coorte de 2015-2020.^12^ A comparação dos resultados de cobertura vacinal com outros estudos é difícil, pois a maior parte apresenta seus resultados para cada uma das vacinas e não para o conjunto das vacinas. Outro fator que dificulta a comparação com estudos internacionais é o número diferente de vacinas disponíveis em cada país. 

No presente estudo, Sete Lagoas/MG foi o município com maior cobertura com doses aplicadas (79%), 2,3 vezes maior que Natal/RN (37%). Nos Estados Unidos, também em crianças nascidas em 2017-2018, a variação observada entre os estados foi 1,5 vez entre a menor cobertura em Oklahoma (58%), e a maior, em Massachusetts (85%)^14^. Na Inglaterra, a cobertura para a maioria das vacinas em 2021 foi mais homogênea, ficando acima de 90% e abaixo da meta de 95% em quase todas as regiões, com exceção de Londres, onde as coberturas de todas as vacinas recomendadas esteve abaixo de 90%, chegando a 82% para meningocócica B (que faz parte da vacinação de rotina naquele país) e a 83% para a tríplice viral.^13^

No presente estudo, 6% das crianças não receberam qualquer dose de vacina, enquanto no México, em 2021, menos de 1% recebeu qualquer dose;^15^ nos Estados Unidos, em 2020, o percentual foi semelhante.^12^

O atraso vacinal é um fator que impacta a cobertura vacinal. As crianças que não tiveram atraso até os 6 meses de idade apresentaram cobertura 31,5% maior comparadas às que tiveram pelo menos um atraso. A comparação dos resultados de atraso vacinal com outros artigos disponíveis na literatura também é difícil, pois geralmente se compara o atraso de cada vacina, e não para o conjunto das vacinas que deveriam ser aplicadas no mesmo período. Na Índia,^16^ foi observado 23,1% de atraso na BCG e 29,3% na primeira dose da DTP, superiores ao observado no presente estudo (6,1% e 11,5%, respectivamente) .

Na Argentina, em 2008,^17^ observou-se 21,2% de atraso para a tríplice viral, e de 37,7% do presente estudo; por outro lado, o atraso na BCG foi quase duas vezes superior: 12,1%.Nas Filipinas, em 2016, além de 25,6% de atraso para BCG, observou-se 34,0% de atraso para a primeira dose e de 50,7% para a segunda dose de pneumocócica,^18^ superiores aos do presente inquérito (5,9% e 10,3%, respectivamente). 

No presente estudo, a vacina BCG apresentou a maior cobertura, única que atingiu a meta recomendada pelo PNI (90%)^1^. Em 2020, a cobertura vacinal para as Américas foi de 94% para BCG, 76% para hepB ao nascer, 85% para a terceira dose da VIP, 73% para a segunda dose da VRH; 84% para a terceira dose da pneumocócica; 90% para a primeira e 72% para a segunda dose da tríplice viral.^19^ Em relação ao presente inquérito, a Organização Pan-Americana da Saúde (OPAS)^19^ mostrou coberturas maiores para BCG, semelhantes para a primeira dose da tríplice viral e terceira dose da pneumocócica (primeiro reforço), e inferiores para hepB ao nascer, terceira dose da VIP, segunda dose da VRH e segunda dose da tríplice viral. 

Em um inquérito realizado no Canadá em 2017,^20^ a cobertura (esquema completo) da VIP (90,7%) foi maior que a constatada no presente estudo. A cobertura para tríplice viral foi semelhante (90,2%), enquanto as coberturas das vacinas pentavalente (75,8%), varicela (82,9%), meningocócica C (87,6%), pneumocócica (81,4%) e rotavírus (78,8%) foram inferiores. 

A cobertura completa com doses aplicadas foi significativamente diferente de acordo com a escolaridade da mãe, evidenciando coberturas menores entre os filhos de mães sem instrução formal ou com ensino fundamental incompleto. O mesmo achado foi observado no Nepal,^15^ nas Filipinas e na Índia.^21^

No estrato A, a cobertura completa com doses aplicadas foi menos de 40% entre as crianças com mães menos escolarizadas, o que, na ponderação, pode ter impactado negativamente a cobertura vacinal nesse estrato.

Os resultados do presente estudo devem ser considerados à luz de suas limitações. Foram incluídas áreas urbanas das capitais, do Distrito Federal e de 12 municípios do interior. Apesar de não ser possível realizar extrapolação de dados para todo o país, a amostra representa importante parcela populacional. O acesso do entrevistador às famílias foi dificultado pela insegurança urbana, pela pandemia de covid-19 e pelo desinteresse em participar, sobretudo nas famílias dos estratos socioeconômicos mais altos. É importante ressaltar que o efeito dessas perdas foi minimizado pelo cálculo dos pesos amostrais.

A leitura da caderneta de vacinação digitalizada também foi dificultada, pois falta padronização na forma de anotar, além de registros de datas ilegíveis e erros de anotação. Quando não foi apresentada a caderneta de vacinação no momento da entrevista e não foi possível recuperá-la, as crianças foram consideradas não vacinadas, o que pode subestimar a cobertura vacinal. A magnitude e abrangência geográfica da amostra e a coleta das datas de aplicação das vacinas diretamente na caderneta de vacinação fortalecem a robustez dos achados. 

As coberturas vacinais estão aquém do esperado, e o PNI do Brasil necessita de ações, em todos os níveis de governo, para retomar os altos níveis alcançados em décadas passadas. Algumas ações devem ser de caráter nacional e outras considerar a realidade de cada local. A identificação de faltosos e uma busca ativa eficiente são imprescindíveis para reduzir o atraso das vacinas e melhorar a cobertura. A simultaneidade da aplicação das vacinas em uma mesma sessão reduz o número de visitas e facilita o cumprimento do calendário por parte do responsável pela criança. 

Os dados apresentados referem-se à coorte de nascidos vivos em 2017 e 2018 e podem refletir realidade diferente da atual para a mesma faixa etária, visto que crianças nascidas no período pandêmico podem ter situação vacinal e modelos explicativos diferentes. Por essa razão, a realização periódica de inquéritos vacinais é fundamental para propiciar medidas para a melhoria das coberturas vacinais.
